# Dr. William Caldwell

**Published:** 1914-06

**Authors:** 


Dr. William Caldwell, Victoria College of Cobourg, 1874; died at his home in Peterborough, Ont., February 8, aged 72. He devoted special attention to ophthalmology and oto-laryngology. He had been physician to the County Jail.

